# A Pilot Randomized Study of NAT2 and YEATS4 Pharmacogenetic Return of Results in Adults with Hypertension and African Ancestry: The GUARDD‐US PGx Study

**DOI:** 10.1002/cpt.70527

**Published:** 2026-10-18

**Authors:** Philip E. Empey, Nita A. Limdi, Dinushika Mohottige, Kady‐Ann Steen‐Burrell, Hrishikesh Chakraborty, Khoa A. Nguyen, Alexander S. Parker, Emily J. Cicali, Girish N. Nadkarni, Michelle A. Ramos, Sabrina Clermont, Erica N. Elwood, Mimsie Robinson, Nandini Shroff, Marc B. Rosenman, Ronald Huang, Larisa H. Cavallari, Julie A. Johnson, Paul R. Dexter, Todd C. Skaar, Kerri L. Cavanaugh, Christina M. Wyatt, Carol R. Horowitz, Rhonda M. Cooper‐DeHoff, Michael T. Eadon, the IGNITE Pragmatic Trials Network

**Affiliations:** ^1^ Department of Pharmacy & Therapeutics University of Pittsburgh School of Pharmacy Pittsburgh Pennsylvania USA; ^2^ Department of Neurology, Heersink School of Medicine University of Alabama at Birmingham Birmingham Alabama USA; ^3^ Barbara T. Murphy Division of Nephrology, Department of Medicine Icahn School of Medicine at Mount Sinai New York New York USA; ^4^ Department of Population Health Science and Policy Icahn School of Medicine at Mount Sinai New York New York USA; ^5^ Duke Clinical Research Institute Duke University School of Medicine Durham North Carolina USA; ^6^ Department of Pharmacotherapy and Translational Research, Center for Pharmacogenomics and Precision Medicine University of Florida College of Pharmacy Gainesville Florida USA; ^7^ University of Florida College of Medicine–Jacksonville Jacksonville Florida USA; ^8^ The Windreich Department of Artificial Intelligence and Human Health Icahn School of Medicine at Mount Sinai New York New York USA; ^9^ The Hasso Plattner Institute for Digital Health at Mount Sinai New York New York USA; ^10^ Icahn School of Medicine at Mount Sinai The Charles Bronfman Institute for Personalized Medicine New York New York USA; ^11^ Icahn School of Medicine at Mount Sinai Institute for Health Equity Research New York New York USA; ^12^ Bethel Gospel Assembly New York New York USA; ^13^ The Institute for Family Health New York New York USA; ^14^ Northwestern University Feinberg School of Medicine Chicago Illinois USA; ^15^ Colleges of Medicine and Pharmacy and Clinical and Translational Science Institute The Ohio State University Columbus Ohio USA; ^16^ Department of Medicine Indiana University School of Medicine Indianapolis Indiana USA; ^17^ Division of Nephrology & Hypertension, Department of Medicine Vanderbilt University Medical Center and Geriatric Research Education and Clinical Center (GRECC) and the Veterans' Wellbeing through Innovation, Systems Science and Experience in Learning Health Systems (VETWISE‐LHS) Nashville Tennessee USA; ^18^ Department of Veteran Affairs Tennessee Valley Healthcare System Nashville Tennessee USA; ^19^ Department of Medicine, Division of Nephrology Duke University School of Medicine Durham North Carolina USA; ^20^ Department of Medicine Icahn School of Medicine at Mount Sinai New York New York USA

## Abstract

Almost half of individuals with hypertension fail to achieve recommended blood pressure (BP) goals. Pharmacogenetic gene‐drug interactions may alter the efficacy of antihypertensive agents. We conducted a multicenter randomized controlled sub‐study of participants (*N* = 1874) with hypertension and self‐reported African ancestry to determine whether pharmacogenetic return of results (RoR) would improve systolic blood pressure (SBP) at 3 months. Participants were randomized to immediate pharmacogenetic RoR (intervention) or delayed RoR at trial completion (control). Clinical decision support conveyed recommendations for *YEATS4*‐guided thiazide diuretic selection and *NAT2*‐guided hydralazine dosing. Prespecified subgroup analyses included those with uncontrolled BP (baseline SBP ≥140 or DBP ≥90 mm Hg) and actionable genotypes. Immediate RoR did not improve SBP at 3 months in a broad population of hypertensive adults with controlled and uncontrolled SBP (covariate‐adjusted change in SBP between groups: −0.3 ± 0.74 mm Hg, 95% CI: −1.8, 1.2). In participants with an actionable *YEATS4* genotype (*N* = 867) predicting reduced thiazide efficacy, immediate RoR led to greater SBP reduction at 3 months (−2.46 mm Hg, 95% CI: −4.57 to −0.36). In participants with uncontrolled hypertension at baseline (*N* = 659), immediate RoR led to a greater reduction in SBP (−2.9 ± 0.74 mm Hg, 95% CI: −5.6, −0.3). Although no SBP reduction was observed in the whole cohort, participants with an actionable *YEATS4* genotype or uncontrolled BP who received immediate RoR had a significantly greater SBP improvement than those with delayed RoR. Future studies of antihypertensive pharmacogenetics should recruit individuals with uncontrolled BP and actionable genotypes.

**Study Highlights**

**WHAT IS THE CURRENT KNOWLEDGE ON THE TOPIC?**

While evidence supporting the implementation of pharmacogenetic testing is growing, evidence on its potential to guide antihypertensive drug therapy is limited. The GUARDD‐US trial provides a unique opportunity to evaluate the effectiveness of returning pharmacogenetic results to improve blood pressure (BP) control via tailored antihypertensive prescribing.

**WHAT QUESTION DID THIS STUDY ADDRESS?**

In a randomized sub‐study of 1874 participants, the GUARDD‐US PGx study sought to understand whether immediate or delayed return of pharmacogenetic results improved systolic blood pressure (SBP) among participants with documented hypertension and self‐reported African ancestry. Two drug‐gene pairs were tested: *YEATS4* for thiazide diuretics and *NAT2* for hydralazine.

**WHAT DOES THIS STUDY ADD TO OUR KNOWLEDGE?**

Immediate return of *YEATS4* and *NAT2* pharmacogenetic results did not lead to greater SBP improvement at 3 months in a population of hypertensive adults. However, in a subgroup of those with an actionable YEATS4 genotype, the immediate return of pharmacogenetic results led to a significant improvement in SBP at 3 months. Immediate return of pharmacogenetic results also improved SBP at 3 months in a prespecified subgroup analysis of participants with uncontrolled hypertension at baseline (SBP ≥140 mm Hg or diastolic BP ≥90 mm Hg).

**HOW MIGHT THIS CHANGE CLINICAL PHARMACOLOGY OR TRANSLATIONAL SCIENCE?**

For optimal impact of return of pharmacogenetic results, future studies of antihypertensive pharmacogenetics should focus on recruiting individuals with uncontrolled BP and actionable genotypes. Further study of implementation of *YEATS4* genotyping to predict thiazide response in hypertensive adults with African ancestry is warranted.

Forty‐eight percent of United States (US) adults have hypertension, with the highest prevalence seen among non‐Hispanic Black adults (58.0%), who are also less likely to receive aggressive therapy.^1^, ^2^ Although antihypertensive use and blood pressure control have improved among non‐Hispanic Black adults in the 2023 National Health and Nutrition Examination Survey (NHANES) survey, blood pressure control is still only achieved in 62.6% of treated Black adults.^3^

Controlling blood pressure requires a multimodal approach spanning lifestyle changes and medications. The previous (2017) American College of Cardiology/American Heart Association (ACC/AHA) High Blood Pressure guideline recommended that Black adults with hypertension but without heart failure or chronic kidney disease (CKD), including those with diabetes mellitus (DM), receive initial antihypertensive treatment including a thiazide‐type diuretic or calcium channel blocker (CCB). Although the new (2025) ACC/AHA guidelines removed race,^4^ the prior guidance continues to influence patients' regimens. Medication response is a complex phenotype, influenced by clinical, environmental, lifestyle, and genetic factors. Prior studies have identified the influence of genetic variation in *YEATS4* and *NAT2* on blood pressure response in patients prescribed thiazide diuretics and hydralazine, respectively.^5^, ^6^, ^7^ Both drug classes are among medications routinely prescribed in patients with self‐described African ancestry.^8^ Approximately 50% of Black individuals in the US are carriers of the *YEATS4* expression quantitative trait locus rs7297610 *T* allele, which is associated with reduced *YEATS4* expression and reduced response to a thiazide diuretic.^6^ N‐acetyltransferase 2 (*NAT2*) is responsible for polymorphic acetylation of hydralazine. Approximately 50% of Black individuals are predicted to be NAT2 normal or intermediate metabolizers with reduced hydralazine efficacy; in contrast, NAT2 poor metabolizers have higher circulating concentrations of hydralazine, leading to increased efficacy and/or a higher likelihood of adverse events like drug‐induced lupus.^8^ These associations have not been prospectively evaluated in the clinical trial setting.

The Genetic Testing to Understand and Address Renal Disease Disparities Across the United States (GUARDD‐US) trial was a pragmatic, randomized controlled trial that evaluated the effect of immediate vs. delayed return of *APOL1* genetic testing results on blood pressure control among patients with hypertension and self‐reported Black race or African ancestry.^9^ Randomized participants with an *APOL1* high risk (APOL1‐HR) genotype were included in the primary analysis of the GUARDD‐US study, which failed to demonstrate a significant effect of immediate vs. delayed return of genotype results on systolic blood pressure (SBP), but did demonstrate a significant benefit in participants with uncontrolled BP at baseline.^9^ Individuals randomized to immediate return of results (RoR) who had one or zero *APOL1* risk alleles were re‐randomized into a pharmacogenetic (PGx) sub‐study to receive immediate or delayed (by 6 months) *YEATS4* and *NAT2* genotyping results. Neither the *YEATS4*‐thiazide nor *NAT2*‐hydralazine drug‐gene pairs had Clinical Pharmacogenetics Implementation Consortium (CPIC) guidelines at the start of GUARDD‐PGx. Both drug‐gene pairs were rated at level 3 evidence (low level of evidence) by ClinPGx (previously PharmGKB).^10^ Thus, the GUARDD‐US investigators saw an opportunity to generate additional data for these drug‐gene pairs within a relevant group of African ancestry participants.

The objective of this randomized controlled trial (GUARDD‐US PGx) was to determine whether PGx test RoR to participants and healthcare professionals improves SBP reduction from baseline to 3 months. Changes in medication prescribing were also assessed.

## METHODS

### Study design

GUARDD‐US PGx (NCT06748040) was a multisite, pragmatic, randomized controlled trial that was a sub‐study of the GUARDD‐US trial (NCT04191824). GUARDD‐US evaluated the effect of return of *APOL1* genetic testing results on blood pressure control among 6754 adults of self‐reported Black race or African ancestry with hypertension. It was approved by a single Institutional Review Board (Duke University, IRB Pro00102979) and all participants provided written informed consent. The trial design was described previously.^11^ The protocol can be found in Supplement S1.

### Participants

Participants were recruited between July 10, 2020 and September 13, 2023 at eight health systems (**Figure** **S1**) that were participating in the GUARDD‐US trial. GUARDD‐US inclusion criteria comprised individuals with hypertension and self‐reported Black race or African ancestry who had (1) neither DM nor CKD or (2) CKD stage 1–4 with or without DM. Exclusion criteria included terminal illness or prior transplantation of the liver, kidney, or bone marrow (allogeneic). Participants who were in the GUARDD‐US immediate genotyping group and who had zero or one *APOL1* risk alleles were then randomized for GUARDD‐US PGx. Participants were not required to be prescribed a thiazide diuretic or hydralazine to be eligible for the study.

### Randomization and blinding

Sub‐study participants were randomized at a 1:1 allocation to immediate PGx RoR (intervention) where testing and RoR are performed immediately or delayed PGx RoR (control) where testing and RoR are conducted at 6 months, stratified by clinical site with random block sizes of four and eight within each site. Participants, providers, and study personnel are not blinded to randomization assignments since genotype results are deposited in EHRs. Investigators were blinded to interim analyses and outcomes during the study.

### Sample size and power

GUARDD‐PGx was a sub‐study of GUARDD‐US and did undergo a formal power assessment. The initial GUARDD‐US study sample size of 954^9^ was determined based on 80% power to detect a difference of 3.5 mm Hg in SBP change from baseline to 3 months between groups with a standard deviation of 18.1 mm Hg (from the GUARDD pilot study).^12^ The effect size is similar to that of lifestyle and pharmacologic interventions in CKD^13^, ^14^ that are known to reduce major adverse cardiovascular events. Based on the actual standard deviation of 19.0 and sample size of 1874, the GUARDD‐PGx power was greater than 95% to detect a 3.5 mm Hg difference between groups.

### Intervention

The intervention was PGx RoR for *YEATS4* and *NAT2* to participants and healthcare professionals. Genotyping was conducted in College of American Pathologists (CAP) accredited and CLIA (Clinical Laboratory Improvement Amendments)‐certified laboratories using a custom polymerase chain reaction (PCR) based TaqMan™ assay (ThermoFisher, Waltham, MA). Assays interrogated a single nucleotide variant (SNV) in *YEATS4 (*rs7297610)^6^ and four SNVs in *NAT2* (rs1799931, rs1801279, rs1799930, rs1801280).^15^ PGx reports included diplotypes and predicted phenotypes (expression or metabolizer status) for both genes and were entered into the electronic health record of each participant.

Clinical decision support (CDS) was established at each institution to guide downstream clinician treatment decisions as described previously.^16^ As institutional practices vary, the CDS modalities were set locally and involved fully electronic pop‐up alert and/or manual clinical consult notes with provider messaging. The core content of this CDS was designed centrally by GUARDD‐US investigators with expertise in PGx and informatics, with algorithmic alignment at all sites.^16^ It included prescribing recommendations based on genotype along with reinforcement of blood pressure and CKD recommendations from the parent study (**Table** **S1**). Specifically, for *YEATS4*, participants with a *T/T* or *C/T* genotype would be expected to have a reduced response to thiazides and should be treated with a calcium channel blocker as first‐line therapy. For *NAT2*, participants who are rapid acetylators (normal or intermediate metabolizers) would be expected to have reduced response to hydralazine vs. poor metabolizers (those without a *4 allele) who may have increased efficacy or adverse effects. Recommendations for *NAT2* rapid acetylators included increasing dose by 50%–100% or selecting an alternative agent. Recommendations for *NAT2* poor metabolizers included a maximum total daily dose of 200 mg of hydralazine or an alternative agent. Participants received a personalized, patient‐oriented educational document about their results and reviewed the results and the potential clinical implications with clinical research coordinators over the phone. To ensure comprehension, a teach‐back method was employed wherein participants were asked to explain the results and clarifications were provided when needed. At baseline, 3‐month, and 6‐month follow‐up study visits, trained personnel measured SBP and DBP using a standardized process described previously.^11^ Of the three blood pressure readings, the first measurement was discarded, while the second and third were recorded and averaged.

### Outcome measures

The primary outcome was change in SBP at 3 months. Secondary outcomes included change in SBP at 6 months and diastolic blood pressure (DBP) at 3 and 6 months.

Three prespecified subgroup analyses assessed SBP at 3 months; (1) in participants with uncontrolled blood pressure (baseline SBP ≥140 or DBP ≥90 mm Hg) at baseline, and in participants with (2) *YEATS* or (3) *NAT2* PGx actionable genotypes. To provide context, exploratory analyses were also conducted for demographic features and discordant subgroups described below. Surveys were employed at these same timepoints to collect participant medication inventories including dosages. A baseline survey collected additional demographics and perceptions about genetic testing.^9^, ^17^ Number of antihypertensive medications was calculated as a measure of intensity of antihypertensive therapy. Adherence was assessed using a validated self‐report measure, and the frequency of missed doses was collected.^17^ Concordance of PGx‐predicted phenotypes and baseline medications in patients with uncontrolled hypertension was determined to assess “PGx opportunity” as well as downstream prescribing changes. The *YEATS* discordant subgroup was defined as participants with uncontrolled blood pressure at baseline who had either (1) a predicted phenotype of reduced thiazide efficacy and were on a thiazide at baseline or (2) a predicted phenotype of standard thiazide efficacy and were not on a thiazide at baseline. Similarly, the *NAT2* discordant subgroup was defined as participants with uncontrolled blood pressure at baseline who had either (1) a predicted phenotype of reduced hydralazine efficacy and were on hydralazine at baseline or (2) a predicted phenotype of increased hydralazine efficacy and were not on hydralazine at baseline.

### Statistical analysis

An intention‐to‐treat (ITT) population approach was used for all analyses. The ITT population included all 1874 participants with zero or one *APOL1* risk alleles who also had non‐missing PGx test results for both *YEATS4* and *NAT2* predicted phenotypes. Statistical comparisons by treatment groups were performed using two‐sided significance tests. For primary analysis, *P*‐value less than 0.0492 was considered statistically significant to maintain an overall alpha of 0.05 accounting for two interim analyses. A covariate‐adjusted linear regression analysis was used to account for any differences in baseline characteristics between intervention and control groups, which included the randomized group assignment as an explanatory factor and these prespecified covariates: baseline SBP, CKD, age, and sex. Secondary and exploratory analyses are reported with least‐square mean estimates and 95% confidence intervals. The full statistical analysis plan is included as a supplemental file. All analyses were performed using SAS software, version 9.4 (SAS Institute, Inc., Cary, NC).

## RESULTS

### Participants

Of the 6754 participants originally randomized in the GUARDD‐US parent trial, 1874 were re‐randomized within the PGx sub‐study (**Figure** **1**). Baseline demographics (**Table** **1**) were similar to those in the parent study and did not differ between the immediate PGx RoR (*n* = 953) and delayed PGx RoR (*n* = 921) groups. Participants had a mean (±SD) age of 55 (±10.7), were majority female (64%), had African ancestry (100%), self‐reported their race as Black or African American (88.3%), and had greater than a high school education (64.8%). Few (13.6%) had prior genetic testing, but most (62.1%) had positive beliefs about it. Upon enrollment, many participants had uncontrolled blood pressure (39.5%), CKD (25.4%), and DM (21.0%). At baseline, most (91.2%) participants were taking medications to control blood pressure with 37.4%, 29.8%, and 24.0% taking 1, 2, or 3 or more antihypertensive medications, respectively. Approximately 43% of individuals reported non‐adherence to antihypertensive therapy according to baseline surveys.

**Figure 1:**
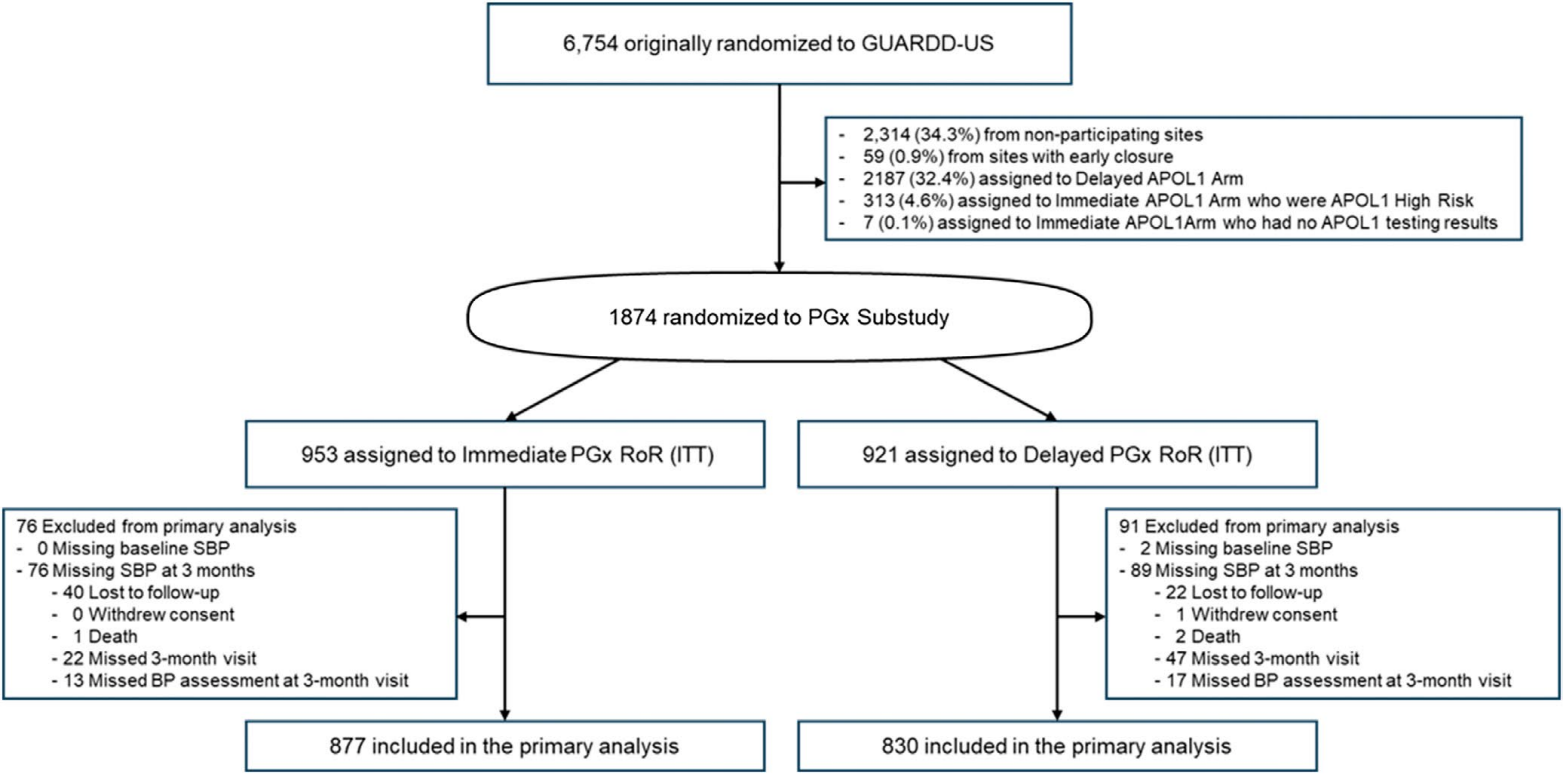
Consort diagram: The primary analysis focused on the change in SBP from baseline to 3 months between treatment groups. Follow‐up continued until 6 months post‐randomization. Participants missing the 3‐ or 6‐month visits were included in analyses if blood pressure was available in the EHR. In the immediate PGx arm, 877 participants were included in the primary analysis as 76 were missing SBP at 3 months. In the delayed PGx arm, 830 participants were included in the primary analysis as 91 were missing either baseline SBP (2) or were missing SBP at 3 months (89).

**Table 1:** Participant characteristics

	Intention‐to‐treat (ITT) participants, *N* = 1874		Uncontrolled blood pressure at baseline, an *a priori specified subgroup, N* = 740	
	Immediate PGx ROR, (Intervention) *N* = 953	Delayed PGx ROR, (Control) *N* = 921	Immediate *PGx* ROR, (Intervention) *N* = 367	Delayed *PGx* ROR, (Control) *N* = 373
Age at randomization (years), mean ± SD	55.3 (10.40)	54.7 (10.97)	54.0 (10.77)	54.0 (10.99)
Sex at birth, no. (%)				
Female	610/953 (64.0%)	590/921 (64.1%)	226/367 (61.6%)	236/373 (63.3%)
Male	343/953 (36.0%)	331/921 (35.9%)	141/367 (38.4%)	137/373 (36.7%)
Self‐Identified Race/Ethnicity, no. (%)				
American Indian, Native American or Alaska Native	2/953 (0.2%)	0/921 (0.0%)	1/367 (0.3%)	0/373 (0.0%)
Asian	1/953 (0.1%)	0/921 (0.0%)	1/367 (0.3%)	0/373 (0.0%)
Black or African American	840/953 (88.1%)	815/921 (88.5%)	333/367 (90.7%)	326/373 (87.4%)
Native Hawaiian or Other Pacific Islander	2/953 (0.2%)	0/921 (0.0%)	1/367 (0.3%)	0/373 (0.0%)
White or European American	0/953 (0.0%)	0/921 (0.0%)	0/367 (0.0%)	0/373 (0.0%)
Middle Eastern or North African/Mediterranean	0/953 (0.0%)	1/921 (0.1%)	0/367 (0.0%)	0/373 (0.0%)
Hispanic/Latino(a)	13/953 (1.4%)	16/921 (1.7%)	4/367 (1.1%)	3/373 (0.8%)
Multiple	77/953 (8.1%)	73/921 (7.9%)	22/367 (6.0%)	33/373 (8.8%)
Other, unknown, prefer not to answer	18/953 (1.9%)	16/921 (1.7%)	5/367 (1.4%)	11/373 (2.9%)
Education—more than high school, no./total no. (%)	623/953 (65.4%)	586/921 (63.6%)	236/366 (64.5%)	234/372 (62.9%)
Household Income < $40,000/year, no./total no. (%)	384/748 (51.3%)	385/738 (52.2%)	158/297 (53.2%)	173/307 (56.4%)
Insurance, no./total no. (%)				
No insurance	20/953 (2.1%)	14/917 (1.5%)	10/367 (2.7%)	4/372 (1.1%)
Medicaid^a^	358/953 (37.6%)	343/917 (37.4%)	138/367 (37.6%)	138/372 (37.1%)
Medicare^a^	122/953 (12.8%)	109/917 (11.9%)	52/367 (14.2%)	44/372 (11.8%)
Medicare and Medicaid^a^	236/953 (24.8%)	249/917 (27.2%)	90/367 (24.5%)	108/372 (29.0%)
Private health insurance only	138/953 (14.5%)	122/917 (13.3%)	48/367 (13.1%)	47/372 (12.6%)
Other insurance (including Military health care/VA)	79/953 (8.3%)	80/917 (8.7%)	29/367 (7.9%)	31/372 (8.3%)
Clinical group				
Mount Sinai	428/953 (44.9%)	412/921 (44.7%)	173/367 (47.1%)	192/373 (51.5%)
Indiana University/University of Pittsburgh	310/953 (32.5%)	296/921 (32.1%)	106/367 (28.9%)	94/373 (25.2%)
University of Florida/University of Alabama Birmingham	215/953 (22.6%)	213/921 (23.1%)	88/367 (24.0%)	87/373 (23.3%)
Poverty^b^, no./total no. (%)	228/747 (30.5%)	230/737 (31.2%)	103/297 (34.7%)	105/307 (34.2%)
Current tobacco use, no./total no. (%)	231/953 (24.2%)	211/920 (22.9%)	99/367 (27.0%)	96/373 (25.7%)
Diabetes Diagnosis^c^, no./total no. (%)	184/898 (20.5%)	183/849 (21.6%)	72/349 (20.6%)	67/339 (19.8%)
Chronic Kidney Disease Diagnosis, no./total no. (%)^c^	243/896 (27.1%)	199/846 (23.5%)	89/349 (25.5%)	66/340 (19.4%)
Baseline blood pressure				
Systolic blood pressure (mm Hg), mean ± SD	133.4 (18.92)	133.3 (19.19)	150.2 (16.75)	150.1 (16.03)
Diastolic blood pressure (mm Hg), mean ± SD	82.9 (13.21)	83.3 (13.05)	93.8 (11.99)	93.7 (11.70)
Systolic blood pressure ≥ 140 mm Hg, no. (%)	285/953 (29.9%)	291/919 (31.7%)	285/367 (77.7%)	291/373 (78.0%)
Diastolic blood pressure ≥ 90 mm Hg, no. (%)	253/953 (26.5%)	253/919 (27.5%)	253/367 (68.9%)	253/373 (67.8%)
Systolic blood pressure < 140 and DBP <90 mm Hg, no.(%)	586/953 (61.5%)	546/919 (59.4%)	0/367 (0.0%)	0/373 (0.0%)
Blood pressure medication use at enrollment^d^, no. (%)	862/953 (90.5%)	847/921 (92.0%)	327/367 (89.1%)	337/373 (90.3%)
Had previous genetic testing, no./total no. (%)	137/940 (14.6%)	114/907 (12.6%)	43/363 (11.8%)	46/370 (12.4%)
Positive beliefs toward PGx testing, no./total no. (%)	571/908 (62.9%)	528/863 (61.2%)	212/354 (59.9%)	198/348 (56.9%)

Percentages are based on the total number of patients with non‐missing response per question.

^a^ These insurance categories identify participants who have “Medicare + Medicaid,” “Medicaid,” or “Medicare” as their health insurance, regardless of any additional coverage.

^b^ Poverty level is defined according to the Federal Poverty Guideline for 2020.

^c^ Defined using Electronic Health Record (EHR) data.

^d^ On blood pressure medication at baseline per Medication Inventory.

### Thiazide and hydralazine use and PGx actionability

About one third (36.8%) of participants reported current thiazide therapy, and only 2.9% reported hydralazine therapy at baseline. Within the prespecified subgroup of participants with uncontrolled hypertension at baseline, no differences in baseline antihypertensive use were observed between the randomized groups. Genotype allele frequencies (**Table** **S2**) were similar between groups. *YEATS4*‐associated reduced thiazide efficacy and *NAT2*‐associated reduced hydralazine efficacy were identified in 51% and 53.2% of participants, respectively. PGx RoR occurred at a median of 21 days (IQR: 17–27) in the immediate RoR arm.

### Primary and secondary blood pressure outcomes

The baseline SBP was not different between the immediate RoR arm (133.4 ± 18.9) and the delayed RoR arm (133.3 ± 19.2). The primary outcome was available in 877 (92.0%) and 830 (90.1%) participants in the immediate and delayed groups, respectively. Overall, the change in SBP (**Table** **2**) was not different at 3 months (immediate: −3.7 ± 19.0 mm Hg vs. delayed: −3.1 ± 19.0 mm Hg, *P* = 0.49). The covariate‐adjusted change in SBP similarly did not show a difference between groups (−0.2 ± 0.78 mm Hg, 95% CI: −1.7, 1.4).

**Table 2:** Change in systolic blood pressure and diastolic blood pressure over 3 and 6 months

	Intention‐to‐treat (ITT) participants, *N* = 1874		*P*‐value
	Immediate PGx ROR, (Intervention)	Delayed PGx ROR, (Control)	
Systolic blood pressure, mean mm Hg (SD), no.			
Baseline	133.4 (18.9), 953	133.3 (19.2), 919	0.8498
3 months	129.6 (17.6), 877	129.8 (18.6), 832	
Change from Baseline at 3 months	−3.7 (19.0), 877	−3.1 (19.0), 832	0.4852
6 months	130.0 (18.5), 837	129.6 (18.1), 811	
Change from Baseline at 6 months	−3.1 (20.0), 837	−3.6 (18.4), 811	0.5860
Diastolic blood pressure, mean mm Hg (SD), no.			
Baseline	82.9 (13.2), 953	83.3 (13.1), 919	0.5746
3 months	80.7 (12.5), 877	80.9 (12.2), 832	
Change from Baseline at 3 months	−2.0 (12.6), 877	−2.1 (11.8), 832	0.8995
6 months	80.7 (12.1), 837	80.8 (13.0), 811	
Change from Baseline at 6 months	−2.1 (12.6), 837	−2.2 (11.9), 811	0.9664

There were also no significant differences in change in SBP at 6 months (immediate: −3.1 ± 20.0 vs. delayed: −3.6 ± 18.4; *P* = 0.5860), change in DBP at 3 (immediate: −2.0 ± 12.6 vs. delayed: −2.1 ± 11.8; *P* = 0.8995) or 6 months (immediate: −2.1 ± 12.6 vs. delayed: −2.2 ± 11.9 *P* = 0.9664), or in overall blood pressure control (<140/90 mmHg) at 3 months (67.3% immediate vs. 68.8% delayed; *P* = 0.5045) or 6 months (67.5% immediate vs. 66.8% delayed; *P* = 0.8888).

### Blood pressure outcomes in those with actionable genotypes and uncontrolled hypertension

Three prespecified subgroup analyses were conducted for the primary outcome of change in SBP at 3 months (**Figure** **2**, **Figure** **S2**). We first assessed change in SBP in those with an actionable *YEATS4* genotype (≥1 *T* allele, reduced *YEATS4* expression). CDS logic encouraged healthcare professionals to start a calcium channel blocker instead of a thiazide as first‐line therapy in those not on a thiazide or add a calcium channel blocker in those already prescribed a thiazide. In participants with a *YEATS4* reduced thiazide efficacy phenotype, the immediate RoR group had a significantly greater decrease in SBP at 3 months. (−4.59 mmHg; 95% CI: −6.34 to −2.83) vs. those in the delayed group (−2.13 mmHg; 95% CI: −3.93 to −0.32) with a mean difference in SBP of −2.46 (95% CI: −4.57 to −0.36; *P* = 0.0048). At 6 months, there was no difference in SBP reduction between groups.

**Figure 2:**
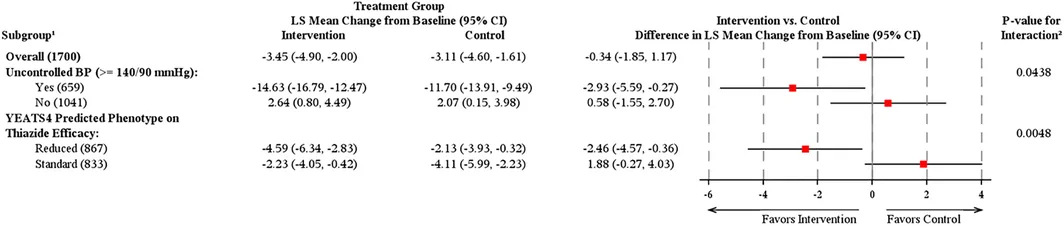
Subgroup analyses of Uncontrolled Blood Pressure and *YEATS4* Actionability. Covariate adjusted linear regression model interactions were observed between the treatment effect and the uncontrolled blood pressure and Reduced Thiazide Efficacy subgroups wherein a significantly greater reduction in systolic blood pressure was observed at 3 months in the intervention group. Forest plots of both statistically significant subanalyses.

We next assessed genotype‐specific effects for *NAT2*. *NAT2* actionability is more challenging to define because genotyping guides the appropriate dosage for hydralazine, not drug selection. Regardless, no significant difference in SBP was observed between groups (NAT2 increased or reduced metabolism) at 3 months. However, the number of participants prescribed hydralazine was <3.5% of the study population at all timepoints.

Finally, we evaluated outcomes in participants with uncontrolled hypertension at baseline (SBP ≥140 mm Hg or diastolic BP ≥90 mm Hg). The immediate RoR group had a larger (−14.6 mm Hg; 95% CI: −16.7 to −12.5) decrease in SBP at 3 months vs. that in the delayed RoR group (−11.7 mm Hg; 95% CI: −13.9 to −9.5). This between‐group difference was −2.9 ± 0.74 mm Hg, 95% CI: −5.6, −0.3, *P* = 0.043). At 6 months, both groups had a similar decrease in SBP. No significant difference in diastolic BP was observed in any of the three prespecified subgroups at 3 or 6 months.

### Effect of intervention on prescribing

Among participants with uncontrolled BP at baseline, a trend toward an increase in antihypertensive medication intensity (number of antihypertensives prescribed) was observed at 6 months (uncorrected *P* = 0.046). The proportion of participants receiving two or more antihypertensive medications in the immediate RoR group increased from 54.8% at baseline to 58.9% at 3 months to 62.0% at 6 months vs. no change in the delayed group (52.3% at baseline, 54.4% at 3 months, and 51.8% at 6 months, **Figure** **3**). Therapy intensification was broadly distributed across medication classes. There were no differences in self‐reported medication non‐adherence between groups.

**Figure 3:**
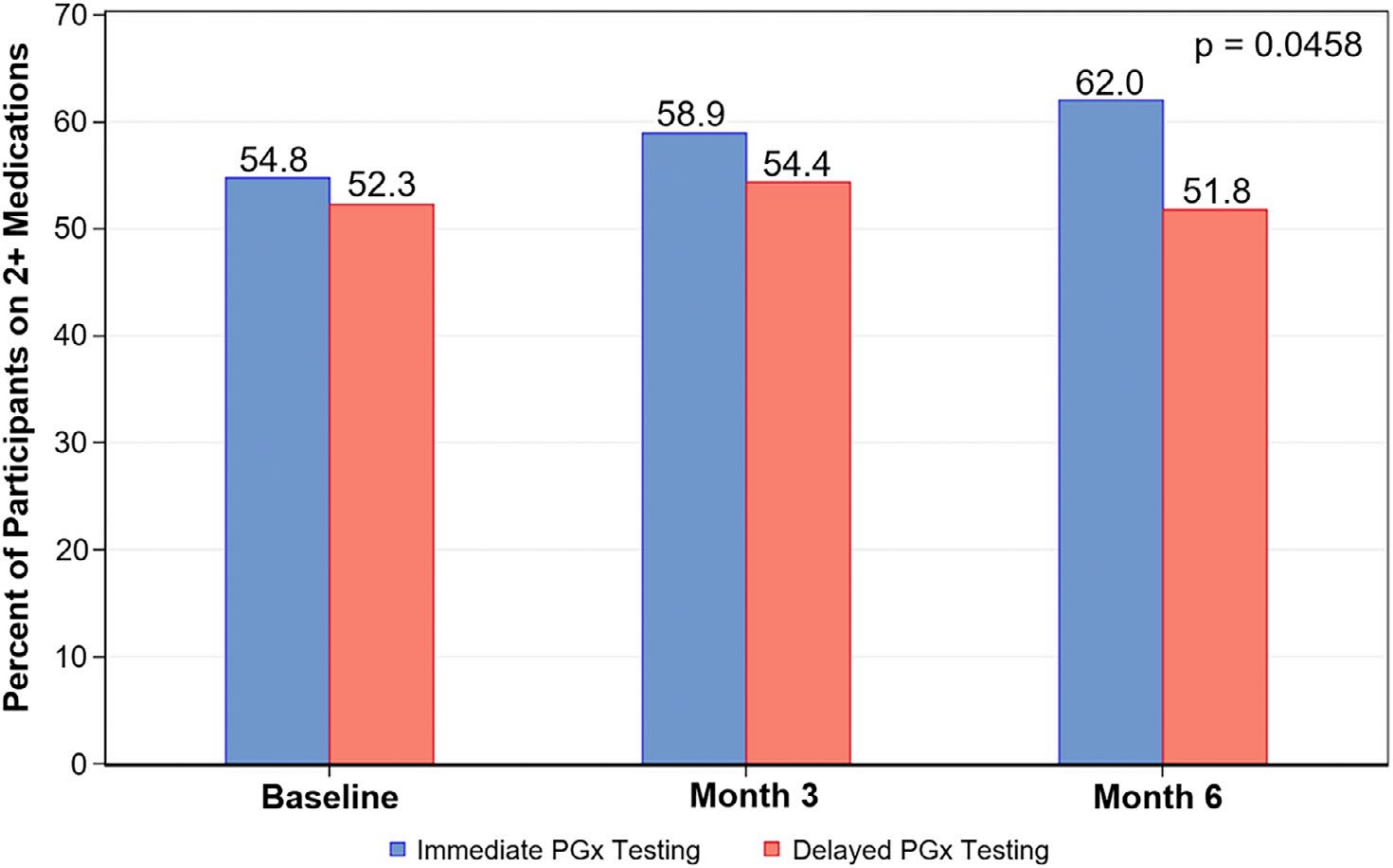
Effect of the intervention on antihypertensive medication prescribing intensity within the uncontrolled hypertension subgroup. The immediate group showed an intensification of antihypertensive therapy over the course of the study as measured by the proportion of participants receiving two or more antihypertensive medications.

### Genotype‐specific concordance

To evaluate PGx effects on clinical outcomes and prescribing, we conducted a set of exploratory analyses of interactions between baseline medication use and PGx results specifically in participants with uncontrolled BP (**Table** **S3**). About one fifth (19.6%, *n* = 368) had drug‐gene discordance with either (1) a YEATS4 reduced thiazide efficacy phenotype and were taking a thiazide or (2) a predicted standard thiazide efficacy and were not taking a thiazide. In these *YEATS4* subgroups (**Figure** **S3**), SBP change did not significantly differ between concordant and discordant groups, although both groups showed a trend toward greater SBP reduction at 3 months. There was no significant change in mean number of antihypertensive medications prescribed between groups (**Table** **S4**), but a trend toward increasing thiazide concordance was observed over the study period (**Table** **S5**).

NAT2 drug‐gene concordance was considered either (1) a NAT2 reduced efficacy phenotype in one taking hydralazine or (2) a predicted increased hydralazine efficacy genotype not taking the drug. Fewer participants (3.8%, *n* = 71) had discordance between hydralazine use and their *NAT2* results. A significant interaction between *NAT2*‐hydralazine discordance and SBP reduction (*P* = 0.034, difference in least‐square mean change from baseline of −11.5 (95% CI: −19.7 to −3.2)) favored improved BP control in the immediate RoR group. However, only 15 participants with uncontrolled BP were prescribed hydralazine at baseline (2%); therefore, this finding was predominantly driven by a small number of participants with reduced hydralazine efficacy who were prescribed an alternative agent.

## DISCUSSION

Black Americans have the highest prevalence of hypertension in the US, yet are least likely to achieve adequate control v. all other racial/ethnic groups.^1^, ^2^ The GUARDD‐US parent study was a large randomized controlled trial designed to evaluate the effect of *APOL1*‐HR RoR to patients and healthcare professionals on blood pressure outcomes. Most participants (85.8%) did not have the APOL1‐HR genotype, and were thus eligible to participate in this embedded pharmacogenomic pilot intervention.^9^ The sub‐study therefore represented an opportunity to pilot a PGx intervention and provide potential value to these participants. We did not demonstrate SBP improvement with the PGx intervention within the overall ITT population, but immediate RoR led to a greater improvement in SBP at 3 months in the prespecified *YEATS4* actionable and the uncontrolled BP subgroups. It is important to note that because GUARDD‐US PGx leveraged the parent trial's infrastructure, it was a pilot study that depended on the parent study population rather than more targeted enrollment of those most likely to benefit from the PGx intervention. It did not exclude participants with controlled hypertension or based on baseline or prior medication use. Results align with expectations, as clinicians may not change antihypertensive therapy for patients with controlled BP. Nonetheless, GUARDD‐US PGx is the largest randomized hypertension PGx study to date. The study demonstrates successful processes for PGx RoR in a pragmatic real‐world environment with a signal of benefit in those with uncontrolled hypertension. Our study population has external validity with similar education levels (64.8% had greater than high school education) and adherence rates (43% non‐adherence) to the general hypertensive population with African ancestry.^18^, ^19^

The implementation of antihypertensive PGx poses unique challenges. Unlike PGx studies of clopidogrel,^20^, ^21^ abacavir,^22^ warfarin,^23^, ^24^, ^25^ or antidepressant therapy,^26^, ^27^ treatment with multiple antihypertensives is common. It is challenging to isolate individual drug‐genotype effects, and monotherapy is not reflective of real‐world clinical care. Non‐pharmacologic factors like salt intake and physical activity also have a significant influence on BP outcomes. Many antihypertensives have unique therapeutic properties related to diseases with guidelines directing their use^28^: like beta‐blockade in heart failure or angiotensin receptor blockade in CKD. Further, it is challenging to account for the enormous variety of prior prescriptions and medication combinations, which may influence a participant's regimen through trial‐and‐error. The goal of hypertension PGx is to optimize an effective and well tolerated antihypertensive regimen.

Few randomized controlled trials of antihypertensive PGx have been conducted, and none in individuals exclusively with African ancestry. Three prior studies prospectively assessed BP in response to PGx. The CKD‐PGX study demonstrated the promise of PGx testing for a panel of 11 antihypertensive gene‐drug pairs in 382 participants, but was not a randomized controlled trial.^7^ Two small clinical trials showed a SBP benefit of antihypertensive PGx‐panel testing among hypertensive individuals in China: the first was a non‐randomized study of 17 genes in 65 participants,^29^ and the second evaluated 10 drug‐gene pairs in 59 participants.^30^ The genes included in these panel studies were culled from the seminal work of consortia like the Pharmacogenomic Evaluation of Antihypertensive Responses (PEAR),^31^ the Genetic Epidemiology of Responses to Antihypertensives study (GERA),^32^ and many others.^33^, ^34^, ^35^, ^36^, ^37^ GUARDD‐US PGx returned recommendations for only two gene‐drug pairs due to a lack of preliminary evidence for other variants in our population.

African ancestry populations are underrepresented in PGx research.^38^, ^39^, ^40^ While race is a social construct, self‐reported ancestry may impact variant frequencies and associated prediction of risk.^41^, ^42^ For example, renin activity varies across populations,^43^ which informs guideline recommendations for drug selection.^28^ We focused GUARDD‐US PGx on two drug‐gene pairs and five variants that had preliminary evidence enabling targeted prospective implementation among African ancestry populations.^5^, ^6^, ^7^ Thiazides remain a first‐line therapy for hypertension, and rs7297610 is an expression quantitative locus in the promoter of *YEATS4*, which reduces thiazide efficacy in African ancestry populations.^6^, ^32^ In GUARDD‐US PGx, over a third of all participants reported baseline thiazide use, and approximately half of participants had a *C/C* genotype. We found that participants with reduced *YEATS4* expression (lower predicted thiazide efficacy) had a greater improvement in SBP with immediate PGx RoR. Presently, there are no Clinical Pharmacogenetics Implementation Consortium (CPIC) guidelines for the *YEATS4*‐thiazide drug‐gene pair; ClinPGx rates this pair as having a low level of evidence supporting the association.^44^ Studies such as GUARDD‐US PGx can be useful for refining the level of evidence for drug‐gene pair implementation.

Hydralazine use is also prevalent in African ancestry populations with heart failure or resistant hypertension,^5^, ^45^ with *NAT2* genetic variation altering hydralazine drug exposure.^46^, ^47^ CPIC guidelines now support *NAT2*‐genotype use as applied in this study.^8^ Rapid and intermediate NAT2 metabolizers are predicted to have reduced hydralazine efficacy, a metabolism status found in half the GUARDD‐US PGx population. Although hydralazine use was lower than expected (3%), we nonetheless identified a signal between hydralazine‐*NAT2* genotype discordance and change in SBP at 3 months.

GUARDD‐PGx tested a set of two monogenic drug‐gene pairs. Hypertension is a polygenic trait.^48^ Likewise, multiple genes affect antihypertensive response including: (1) metabolic polymorphisms affecting drug exposure, (2) receptor polymorphisms affecting pharmacodynamics, and (3) variants uncovered through genome‐wide association studies (GWAS), often with mechanisms that remain to be elucidated.^49^ For example, metoprolol response is affected by polymorphisms in both CYP2D6 enzymes and adrenoreceptors.^50^ It is possible that polygenic models will be required to appropriately dose thiazide diuretics and hydralazine as well.

Genomic implementation has been hindered by a lack of adherence to PGx recommendations and by limited availability of PGx guidance in specific underrepresented subgroups. An observational PGx implementation study recently evaluated CDS actionability in Black Americans following PGx‐panel testing, including *YEATS4* and *NAT2*.^51^ The study found only a modest uptake of actionable PGx recommendations for their broad PGx panel. In GUARDD‐US PGx, we found prescribing intensification among participants receiving immediate PGx RoR at 6 months, broadly distributed across a variety of antihypertensives. However, SBP changes were no longer observed beyond 3 months. The reason for the lack of outcome persistence is unknown but could include a waning impact of the intervention on patient lifestyle modification or medication adherence. Implementation science tools designed to optimize intervention fidelity and adherence to RoR recommendations are essential to realize the promise of PGx and future clinical trials.^52^, ^53^, ^54^ GUARDD‐US PGx also focused on patient‐based RoR in efforts to improve adherence, nutrition, and physical activity.

Our study has additional limitations to consider. First, enrollment criteria and power were determined for the parent GUARDD study, not the PGx sub‐study. GUARDD‐US PGx was pragmatic and included a wide variety of participants: well‐controlled BP, uncontrolled BP, and treatment‐resistant patients. The study was also agnostic to baseline medication usage. Further, the actionable genotypes were highly prevalent without defined CPIC guidance at the study's start. These factors may have constrained actionability and the ability to optimize genotype‐guided medication regimens. Taken together, the level of evidence may not have been strong enough to support a prospective intervention. Second, individual institutions used their preferred methods of RoR CDS. This approach increased feasibility and real‐world fidelity but added heterogeneity. Third, other factors that may impact blood pressure were not measured in the parent GUARDD‐US trial such as lifestyle modification, dietary changes, and concurrent over‐the‐counter medications. Finally, the COVID‐19 pandemic may have affected provider and patient adherence to PGx recommendations, particularly with the ubiquitous use of telehealth in that period. Because recruitment was somewhat slower during the COVID pandemic, we extended our recruitment window by approximately 9 months.

In conclusion, GUARDD‐US PGx represents an important step in understanding antihypertensive PGx implementation and impact in a population disproportionately impacted by adverse clinical outcomes of hypertension. We successfully demonstrated multi‐site real‐world implementation and report a potential benefit in individuals with uncontrolled hypertension and *YEATS4/NAT2* RoR. Future hypertension PGx studies should focus on optimizing reach, effectiveness, and sustainability.

## FUNDING

This work was supported by grants from the National Institutes of Health (U01 HG007269, U01 HG010232, U01 HG010248, U01 HG010231, U01 HG010245, U01 HG010225), and by the NIH IGNITE Network (https://gmkb.org). The content is solely the responsibility of the authors and does not necessarily represent the official views of the National Institutes of Health. Some investigators on the trial were also supported by NIH grants (grants UL1TR002243 and K26128274 (Dr Cavanaugh); grant K23DK139454 (Dr Mohottige)). The National Human Genome Research Institute (NHGRI) sponsored this study and was responsible for appointing an independent data safety and monitoring board and providing funding for the study. The NHGRI did not collect data, manage the study, conduct analysis, or interpret data. Two NHGRI investigators reviewed the final draft of this manuscript, but did not provide approval or make the decision to submit this manuscript for publication.

## CONFLICT OF INTEREST

Dr Cavanaugh reported receiving grants from AstraZeneca outside the submitted work. Dr Mohottige reported serving as a consultant for LabCorps outside the submitted work. Dr Nadkarni reported being cofounder of and holding equity and stock options in Renalytix and Pensieve Health; and receiving personal fees from Renalytix and Vertex Pharma outside the submitted work; in addition, Dr Nadkarni had a patent for risk using genomic and other data licensed. Dr Cavallari reported receiving research support from Werfen outside the submitted work. Dr Johnson reported receiving personal fees from the University of North Carolina at Chapel Hill, Medical University of South Carolina, New York University, University of Mississippi, Mercer University, St Jude Children's Research Hospital, and the University of Cincinnati outside the submitted work. All other authors declared no competing interests for this work.

## AUTHOR CONTRIBUTIONS

P.E.E., N.A.L., D.M., K.‐A.S.‐B., H.C., K.A.N., S.V., R.R., K.L.C., C.M.W., C.R.H., R.M.C.‐D., and M.T.E. wrote the manuscript. K.L.C., E.J.C., E.N.E., S.V., P.R.D., T.C.S., L.H.C., J.A.J., C.R.H., R.M.C.‐D., and M.T.E. designed the research. All authors performed the research. P.E.E., H.C., R.H., and M.T.E. analyzed the data.

## DISCLAIMER

The content is solely the responsibility of the authors and does not necessarily represent the official views of the National Institutes of Health.

## Supporting information


Figure S1.

File S1.

Data S1.
