# Supplementary material for: A Pilot Randomized Study of NAT2 and YEATS4 Pharmacogenetic Return of Results in Adults with Hypertension and African Ancestry: The GUARDD‐US PGx Study
Source: Clin Pharmacol Ther. 2026 Oct 18:10.1002/cpt.70527. Online ahead of print. doi: 10.1002/cpt.70527 (PMC13677980; doi:10.1002/cpt.70527)
Supplement: Supplementary file 2 [file CPT-9999-0-s003.docx]

# SUPPLEMENT 2

## Figure S1: Clinical sites

## Figure S2: Subgroup analyses by randomized treatment group

## Figure S3: Subgroup analyses of baseline medication concordance with PGx against the primary endpoint

## Table S1: Genotype-guided recommendations

| **Goal  (Drug Class)** | **Gene** | **Genotype** | **Predicted Phenotype** | **Recommendation** |
| --- | --- | --- | --- | --- |
| First Line Therapy  (Thiazide diuretic) | *YEATS4* | *C/C* | Standard Thiazide Efficacy | This individual is predicted to have standard thiazide efficacy. Consider use of a thiazide diuretic or calcium channel blocker as first line treatment of hypertension. |
| First Line Therapy  (Thiazide diuretic) | *YEATS4* | *TT* or *TC* | Reduced Thiazide Efficacy | This individual is predicted to have reduced thiazide efficacy. Consider use of an alternate first line therapy such as a calcium channel blocker for treatment of hypertension. |
| Resistant Hypertension  (Hydralazine) | *NAT2* | 1 or more copy of **4* | Reduced Hydralazine Efficacy | This individual is predicted to have reduced hydralazine efficacy and may require a 50-100% higher starting dose or an alternate agent such as clonidine, spironolactone, or doxazosin for resistant hypertension. |
| Resistant Hypertension  (Hydralazine) | *NAT2* | 2 non-**4* alleles | Increased Hydralazine Efficacy | This individual’s NAT2 genetic test result is associated with increased hydralazine levels, which may lead to increased efficacy and/or adverse effects. |

## Table S2: PGx testing results

|  | **All Participants N=1874** | **Immediate PGx ROR N=953** | **Delayed PGx ROR**  **N=921** |
| --- | --- | --- | --- |
| **PGx testing result received in study database** | 1864/1874 (99.5%) | 953/953 (100.0%) | 911/921 (98.9%) |
| **Timing of confirmed return to participants** | 1627/1864 (87.3%) | 888/953 (93.2%) | 739/911 (81.1%) |
| 0 - < 3 months after randomization | 889/1627 (54.6%) | 886/888 (99.8%) | 3/739 (0.4%) |
| 3 - < 6 months after randomization | 3/1627 (0.2%) | 2/888 (0.2%) | 1/739 (0.1%) |
| ≥ 6 months after randomization | 735/1627 (45.2%) | 0/888 (0.0%) | 735/739 (99.5%) |
| **YEATS4 predicted phenotype proportions** | | | |
| YEATS4 reduced thiazide efficacy | 951/1864 (51.0%) | 473/953 (49.6%) | 478/911 (52.5%) |
| YEATS4 standard thiazide efficacy | 913/1864 (49.0%) | 480/953 (50.4%) | 433/911 (47.5%) |
| **NAT2 predicted phenotype proportions** | | | |
| NAT2 reduced hydralazine efficacy | 992/1864 (53.2%) | 508/953 (53.3%) | 484/911 (53.1%) |
| NAT2 increased hydralazine efficacy | 872/1864 (46.8%) | 445/953 (46.7%) | 427/911 (46.9%) |
| **Time of results to primary endpoint assessment** | | | |
| 0 day | 1040/1874 (55.5%) | 122/953 (12.8%) | 918/921 (99.7%) |
| 1 - 29 days | 3/1874 (0.2%) | 3/953 (0.3%) | 0/921 (0.0%) |
| 30 - 59 days | 45/1874 (2.4%) | 45/953 (4.7%) | 0/921 (0.0%) |
| 60 - 89 days | 520/1874 (27.7%) | 518/953 (54.4%) | 2/921 (0.2%) |
| 90 days or more | 266/1874 (14.2%) | 265/953 (27.8%) | 1/921 (0.1%) |

## Table S3: Concordance of baseline medications with PGx results in patients uncontrolled blood pressure.

|  | **All Participants N=1874** | **Immediate PGx ROR N=953** | **Delayed PGx ROR**  **N=921** |
| --- | --- | --- | --- |
| **YEATS4 Discordant Subgroup^1^** | **368/1874 (19.6%)** | **189/953 (19.8%)** | **179/921 (19.4%)** |
| YEATS4 Discordant Subgroup 1^2^ | 126/1874 (6.7%) | 64/953 (6.7%) | 62/921 (6.7%) |
| YEATS4 Discordant Subgroup 2^3^ | 242/1874 (12.9%) | 125/953 (13.1%) | 117/921 (12.7%) |
| **YEATS4 Concordant Subgroup^4^** | **368/1874 (19.6%)** | **178/953 (18.7%)** | **190/921 (20.6%)** |
| YEATS4 Concordant Subgroup 1^5^ | 242/1874 (12.9%) | 113/953 (11.9%) | 129/921 (14.0%) |
| YEATS4 Concordant Subgroup 2^6^ | 126/1874 (6.7%) | 65/953 (6.8%) | 61/921 (6.6%) |
| **NAT2 Discordant Subgroup^7^** | **71/1874 (3.8%)** | **39/953 (4.1%)** | **32/921 (3.5%)** |
| NAT2 Discordant Subgroup 1^8^ | 7/1874 (0.4%) | 5/953 (0.5%) | 2/921 (0.2%) |
| NAT2 Discordant Subgroup 2^9^ | 64/1874 (3.4%) | 34/953 (3.6%) | 30/921 (3.3%) |
| **NAT2 Concordant Subgroup^10^** | **100/1874 (5.3%)** | **53/953 (5.6%)** | **47/921 (5.1%)** |
| NAT2 Concordant Subgroup 1^11^ | 92/1874 (4.9%) | 48/953 (5.0%) | 44/921 (4.8%) |
| NAT2 Concordant Subgroup 2^12^ | 8/1874 (0.4%) | 5/953 (0.5%) | 3/921 (0.3%) |

1. YEATS4 Discordant Subgroup = YEATS4 Discordant Subgroup 1 + YEATS4 Discordant Subgroup 2.
2. YEATS4 Discordant Subgroup 1 = Participants with uncontrolled blood pressure (i.e., SBP ≥ 140 mmHg or DBP ≥ 90 mmHg) at baseline who have reduced thiazide efficacy as the YEATS4 predicted phenotype and were on thiazide medication at baseline.
3. YEATS4 Discordant Subgroup 2 = Participants with uncontrolled blood pressure (i.e., SBP ≥ 140 mmHg or DBP ≥ 90 mmHg) at baseline who have standard thiazide efficacy as the YEATS4 predicted phenotype and were not on thiazide medication at baseline.
4. YEATS4 Concordant Subgroup = YEATS4 Concordant Subgroup 1 + YEATS4 Concordant Subgroup 2.
5. YEATS4 Concordant Subgroup 1 = Participants with uncontrolled blood pressure (i.e., SBP ≥ 140 mmHg or DBP ≥ 90 mmHg) at baseline, who have reduced thiazide efficacy as the YEATS4 predicted phenotype and were not on thiazide medication at baseline.
6. YEATS4 Concordant Subgroup 2 = Participants with uncontrolled blood pressure (i.e., SBP ≥ 140 mmHg or DBP ≥ 90 mmHg) at baseline, who have standard thiazide efficacy as the YEATS4 predicted phenotype and were on thiazide medication at baseline.
7. NAT2 Discordant Subgroup = NAT2 Discordant Subgroup 1 + NAT2 Discordant Subgroup 2.
8. NAT2 Discordant Subgroup 1 = Participants with uncontrolled blood pressure (i.e., SBP ≥ 140 mmHg or DBP ≥ 90 mmHg) at baseline who have reduced hydralazine efficacy as the NAT2 predicted phenotype, had resistant HTN (i.e., on 3 or more Antihypertensive medications at baseline) and who were on hydralazine medication at baseline.
9. NAT2 Discordant Subgroup 2 = Participants with uncontrolled blood pressure (i.e., SBP ≥ 140 mmHg or DBP ≥ 90 mmHg) at baseline who have increased hydralazine efficacy as the NAT2 predicted phenotype, had resistant HTN (i.e., on 3 or more Antihypertensive medications at baseline) and were not on hydralazine medication at baseline.
10. NAT2 Concordant Subgroup = NAT2 Concordant Subgroup 1 + NAT2 Concordant Subgroup 2.
11. NAT2 Concordant Subgroup 1 = Participants with uncontrolled blood pressure (i.e., SBP ≥ 140 mmHg or DBP ≥ 90 mmHg) at baseline, who have reduced hydralazine efficacy as the NAT2 predicted phenotype, had resistant HTN (i.e., on 3 or more antihypertensive medications at baseline) and who were not on hydralazine medication at baseline
12. NAT2 Concordant Subgroup 2 = Participants with uncontrolled blood pressure (i.e., SBP ≥ 140 mmHg or DBP ≥ 90 mmHg) at baseline, who have increased hydralazine efficacy as the NAT2 predicted phenotype, had resistant HTN (i.e., on 3 or more antihypertensive medications at baseline) and who were on hydralazine medication at baseline.

## Table S4: Medications at 0, 3, and 6 months

|  | **Intention-to-treat (ITT) participants, N=1874** | | | | | | **Uncontrolled blood pressure at baseline, an *a priori specified subgroup,* N=740** | | | | | | |
| --- | --- | --- | --- | --- | --- | --- | --- | --- | --- | --- | --- | --- | --- |
|  | **Immediate PGx ROR N=953** | | | **Delayed PGx ROR**  **N=921** | | | **Immediate PGx ROR N=367** | | | **Delayed PGx ROR**  **N=373** | | | |
|  | **Baseline**  **N=953** | **3-month**  **N=879** | **6-month**  **N=811** | **Baseline**  **N=921** | **3-month**  **N=845** | **6-month**  **N=777** | **Baseline**  **N=367** | **3-month**  **N=336** | **6-month**  **N=308** | **Baseline**  **N=373** | **3-month**  **N=333** | | **6-month**  **N=309** |
| **Medication prescription, by drug class** | | | | | | | | | | | |  |  |
| Thiazide | 341 (35.8%) | 321 (36.5%) | 286 (35.3%) | 348 (37.8%) | 315 (37.3%) | 283 (36.4%) | 129 (35.1%) | 120 (35.7%) | 113 (36.7%) | 125 (33.5%) | 110 (33.0%) | | 100 (32.4%) |
| Calcium channel blocker | 517 (54.2%) | 491 (55.9%) | 454 (56.0%) | 476 (51.7%) | 456 (54.0%) | 408 (52.5%) | 207 (56.4%) | 190 (56.5%) | 187 (60.7%) | 194 (52.0%) | 187 (56.2%) | | 169 (54.7%) |
| Hydralazine | 28 (2.9%) | 26 (3.0%) | 20 (2.5%) | 27 (2.9%) | 25 (3.0%) | 21 (2.7%) | 13 (3.5%) | 11 (3.3%) | 7 (2.3%) | 6 (1.6%) | 6 (1.8%) | | 7 (2.3%) |
| Beta blocker | 232 (24.3%) | 216 (24.6%) | 196 (24.2%) | 232 (25.2%) | 221 (26.2%) | 191 (24.6%) | 92 (25.1%) | 87 (25.9%) | 76 (24.7%) | 94 (25.2%) | 89 (26.7%) | | 78 (25.2%) |
| ACE inhibitor | 220 (23.1%) | 208 (23.7%) | 189 (23.3%) | 217 (23.6%) | 206 (24.4%) | 196 (25.2%) | 81 (22.1%) | 76 (22.6%) | 72 (23.4%) | 87 (23.3%) | 80 (24.0%) | | 75 (24.3%) |
| Angiotensin receptor blocker | 214 (22.5%) | 207 (23.5%) | 198 (24.4%) | 222 (24.1%) | 207 (24.5%) | 181 (23.3%) | 84 (22.9%) | 84 (25.0%) | 81 (26.3%) | 93 (24.9%) | 84 (25.2%) | | 72 (23.3%) |
| Alpha blocker | 5 (0.5%) | 6 (0.7%) | 5 (0.6%) | 8 (0.9%) | 11 (1.3%) | 7 (0.9%) | 2 (0.5%) | 3 (0.9%) | 2 (0.6%) | 5 (1.3%) | 8 (2.4%) | | 4 (1.3%) |
| Loop diuretic | 41 (4.3%) | 37 (4.2%) | 34 (4.2%) | 37 (4.0%) | 36 (4.3%) | 34 (4.4%) | 16 (4.4%) | 16 (4.8%) | 14 (4.5%) | 13 (3.5%) | 13 (3.9%) | | 12 (3.9%) |
| K-sparing diuretic | 20 (2.1%) | 18 (2.0%) | 16 (2.0%) | 25 (2.7%) | 27 (3.2%) | 25 (3.2%) | 7 (1.9%) | 7 (2.1%) | 6 (1.9%) | 3 (0.8%) | 5 (1.5%) | | 4 (1.3%) |
| Mineralocorticoid receptor antagonist | 41 (4.3%) | 43 (4.9%) | 41 (5.1%) | 46 (5.0%) | 38 (4.5%) | 33 (4.2%) | 18 (4.9%) | 19 (5.7%) | 18 (5.8%) | 14 (3.8%) | 12 (3.6%) | | 12 (3.9%) |
| Vasodilator | 33 (3.5%) | 31 (3.5%) | 25 (3.1%) | 35 (3.8%) | 34 (4.0%) | 30 (3.9%) | 16 (4.4%) | 14 (4.2%) | 11 (3.6%) | 8 (2.1%) | 10 (3.0%) | | 9 (2.9%) |
| Alpha 2 receptor agonist | 13 (1.4%) | 12 (1.4%) | 11 (1.4%) | 16 (1.7%) | 17 (2.0%) | 15 (1.9%) | 7 (1.9%) | 7 (2.1%) | 7 (2.3%) | 11 (2.9%) | 11 (3.3%) | | 10 (3.2%) |
| Direct renin inhibitor | 0  (0.0%) | 0 (0.0%) | 0 (0.0%) | 0 (0.0%) | 0 (0.0%) | 0 (0.0%) | 0 (0.0%) | 0 (0.0%) | 0 (0.0%) | 0 (0.0%) | 0 (0.0%) | | 0 (0.0%) |
| SGLT2 inhibitor* | 2 (0.2%) | 2 (0.2%) | 5 (0.6%) | 6 (0.7%) | 5 (0.6%) | 6 (0.8%) | 0 (0.0%) | 0 (0.0%) | 0 (0.0%) | 0 (0.0%) | 0 (0.0%) | | 0 (0.0%) |
| **Number of anti-hypertensive medications** | | | | | | | | | | | | | |
| mean±SD | 1.7712 (1.1321) | 1.8214 (1.1228) | 1.8064 (1.1092) | 1.8295 (1.1704) | 1.8805 (1.2008) | 1.8340 (1.2097) | 1.8038 (1.1872) | 1.8631 (1.1714) | 1.9123 (1.1452) | 1.7587 (1.1574) | 1.8468 (1.2163) | | 1.7896 (1.2265) |
| 0 | 91 (9.5%) | 72 (8.2%) | 74 (9.1%) | 74 (8.0%) | 60 (7.1%) | 68 (8.8%) | 40 (10.9%) | 33 (9.8%) | 27 (8.8%) | 36 (9.7%) | 28 (8.4%) | | 33 (10.7%) |
| 1 | 355 (37.3%) | 319 (36.3%) | 279 (34.4%) | 347 (37.7%) | 313 (37.0%) | 289 (37.2%) | 126 (34.3%) | 105 (31.3%) | 90 (29.2%) | 142 (38.1%) | 124 (37.2%) | | 116 (37.5%) |
| 2 | 279 (29.3%) | 270 (30.7%) | 260 (32.1%) | 279 (30.3%) | 259 (30.7%) | 232 (29.9%) | 109 (29.7%) | 112 (33.3%) | 109 (35.4%) | 115 (30.8%) | 100 (30.0%) | | 87 (28.2%) |
| ≥ 3 | 228 (23.9%) | 218 (24.8%) | 198 (24.4%) | 221 (24.0%) | 213 (25.2%) | 188 (24.2%) | 92 (25.1%) | 86 (25.6%) | 82 (26.6%) | 80 (21.4%) | 81 (24.3%) | | 73 (23.6%) |
| **Changes in number of anti-hypertensive medications, Baseline to 3-months, and baseline to 6-months** | | | | | | | | | | | | | |
| +3 |  | 0 (0.0%) | 1 (0.1%) |  | 0 (0.0%) | 0 (0.0%) |  | 0 (0.0%) | 1 (0.3%) |  | 0 (0.0%) | | 0 (0.0%) |
| +2 |  | 5 (0.6%) | 8 (1.0%) |  | 6 (0.7%) | 8 (1.0%) |  | 3 (0.9%) | 6 (1.9%) |  | 4 (1.2%) | | 4 (1.3%) |
| +1 |  | 51 (5.8%) | 60 (7.4%) |  | 51 (6.0%) | 65 (8.4%) |  | 28 (8.3%) | 35 (11.4%) |  | 31 (9.3%) | | 37 (12.0%) |
| 0 (no change) |  | 792 (90.1%) | 691 (85.2%) |  | 764 (90.4%) | 659 (84.8%) |  | 291 (86.6%) | 246 (79.9%) |  | 285 (85.6%) | | 246 (79.6%) |
| -1 |  | 24 (2.7%) | 41 (5.1%) |  | 22 (2.6%) | 39 (5.0%) |  | 10 (3.0%) | 18 (5.8%) |  | 13 (3.9%) | | 19 (6.1%) |
| -2 |  | 5 (0.6%) | 6 (0.7%) |  | 1 (0.1%) | 5 (0.6%) |  | 3 (0.9%) | 1 (0.3%) |  | 0 (0.0%) | | 3 (1.0%) |
| -3 |  | 2 (0.2%) | 4 (0.5%) |  | 1 (0.1%) | 1 (0.1%) |  | 1 (0.3%) | 1 (0.3%) |  | 0 (0.0%) | | 0 (0.0%) |

†p<0.05 (intervention vs. control). *SGLT2 (sodium glucose cotransporter 2) inhibitors were counted as antihypertensives due to their modest blood pressure lowering effect

## Table S5: Changes in medication concordance with PGx results

|  | **Immediate PGx ROR N=953** | **Delayed PGx ROR N=921** |
| --- | --- | --- |
| **YEATS4 Discordant Group** | **189/953 (19.8%)** | **179/921 (19.4%)** |
| **YEATS4 Discordant Subgroup 1^2^ (PP= Reduced Thiazide Efficacy)** | **64/953 (6.7%)** | **62/921 (6.7%)** |
| On thiazide at baseline | 64/64 (100.0%) | 62/62 (100.0%) |
| On thiazide at 3 months | 54/59 (91.5%) | 53/56 (94.6%) |
| On thiazide at 6 months | 49/55 (89.1%) | 49/54 (90.7%) |
| **YEATS4 Discordant Subgroup 2^3^ (PP= Standard Thiazide Efficacy)** | **125/953 (13.1%)** | **117/921 (12.7%)** |
| On thiazide at baseline | 0/125 (0.0%) | 0/117 (0.0%) |
| On thiazide at 3 months | 5/108 (4.6%) | 3/109 (2.8%) |
| On thiazide at 6 months | 7/99 (7.1%) | 4/100 (4.0%) |
| **NAT2 Discordant Group** | **39/953 (4.1%)** | **32/921 (3.5%)** |
| **NAT2 Discordant Subgroup 1^2^ (PP= Reduced Hydralazine Efficacy)** | **5/953 (0.5%)** | **2/921 (0.2%)** |
| On hydralazine at baseline | 5/5 (100.0%) | 2/2 (100.0%) |
| On hydralazine at 3 months | 2/3 (66.7%) | 0/2 (0.0%) |
| On hydralazine at 6 months | 2/3 (66.7%) | 0/1 (0.0%) |
| **NAT2 Discordant Subgroup 2^3^ (PP= Increased Hydralazine Efficacy)** | **34/953 (3.6%)** | **30/921 (3.3%)** |
| On hydralazine at baseline | 0/34 (0.0%) | 0/30 (0.0%) |
| On hydralazine at 3 months | 0/33 (0.0%) | 1/28 (3.6%) |
| On hydralazine at 6 months | 0/30 (0.0%) | 1/25 (4.0%) |
